# Targeting cancer-cell mitochondria and metabolism to improve radiotherapy response

**DOI:** 10.1016/j.tranon.2020.100905

**Published:** 2020-10-14

**Authors:** Emma McCann, Jacintha O'Sullivan, Simone Marcone

**Affiliations:** a Department of Surgery, Trinity Translational Medicine Institute, St. James's Hospital, Trinity College Dublin, Dublin, Ireland; b M.Sc. in Translational Oncology, Trinity College Dublin, Dublin, Ireland

**Keywords:** Mitochondrial dysfunction, Cancer metabolism, Radiotherapy, Radioresistance, Cancer treatment

## Abstract

Radiotherapy is a regimen that uses ionising radiation (IR) to treat cancer. Despite the availability of several therapeutic options, cancer remains difficult to treat and only a minor percentage of patients receiving radiotherapy show a complete response to the treatment due to development of resistance to IR (radioresistance). Therefore, radioresistance is a major clinical problem and is defined as an adaptive response of the tumour to radiation-induced damage by altering several cellular processes which sustain tumour growth including DNA damage repair, cell cycle arrest, alterations of oncogenes and tumour suppressor genes, autophagy, tumour metabolism and altered reactive oxygen species. Cellular organelles, in particular mitochondria, are key players in mediating the radiation response in tumour, as they regulate many of the cellular processes involved in radioresistance. In this article has been reviewed the recent findings describing the cellular and molecular mechanism by which cancer rewires the function of the mitochondria and cellular metabolism to enhance radioresistance, and the role that drugs targeting cellular bioenergetics have in enhancing radiation response in cancer patients.

## Graphical abstract

**Figure:**
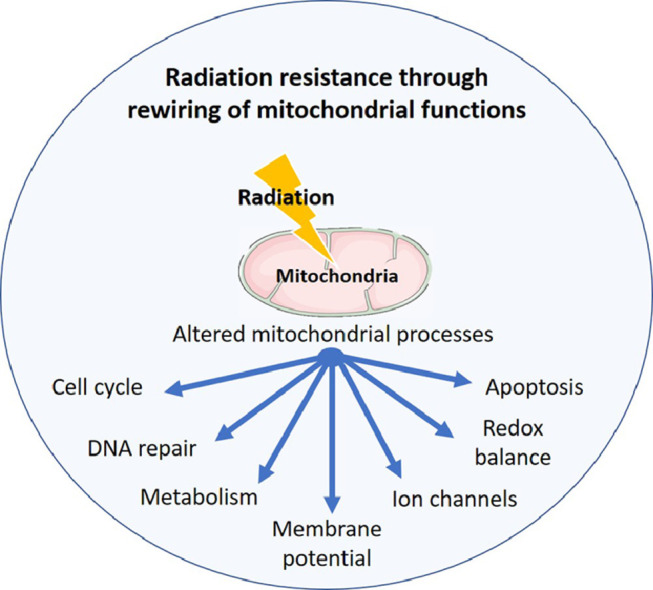

## Introduction

**Fig. 1:**
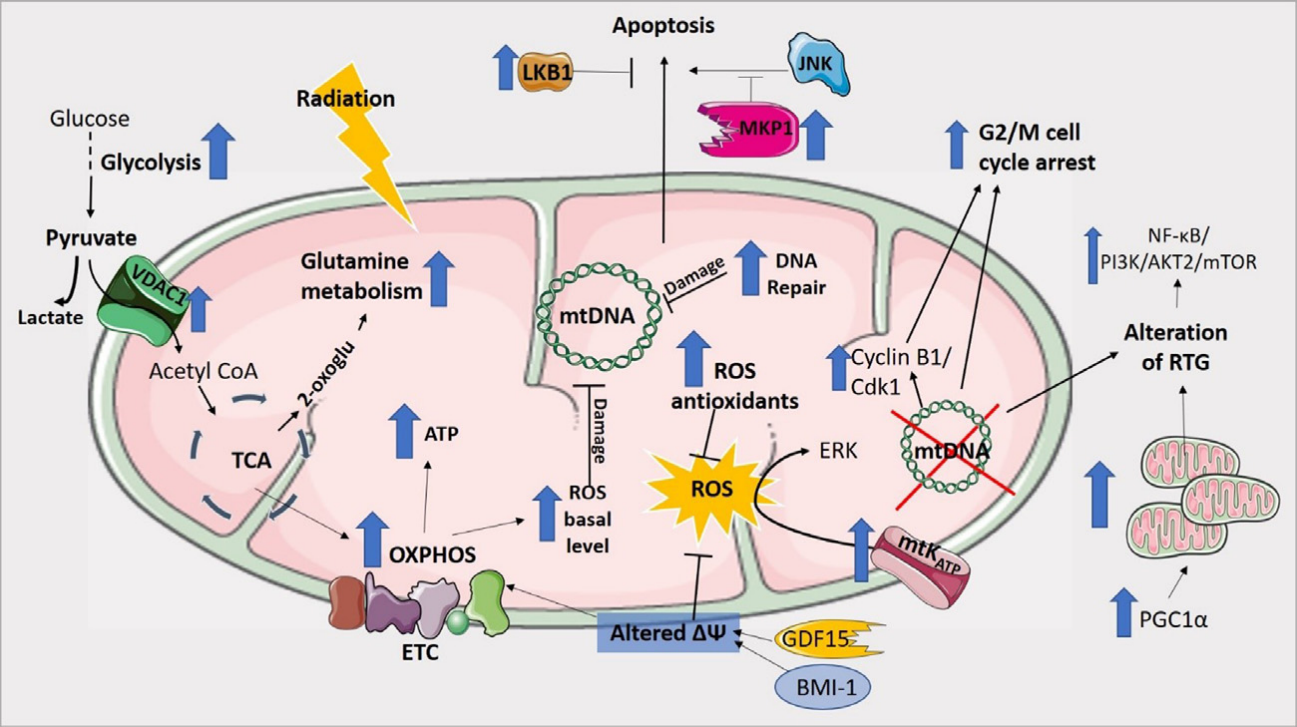
Cellular mechanisms contributing to radioresistance through rewiring of mitochondrial function and metabolism in cancer.

Cancer is a multifactorial disease and the choice of treatment options is dependent on many factors. Therapeutic options include surgery, chemotherapy, immunotherapy, targeted and endocrine therapies, and radiation therapy [1,2]. Despite the availability of these options cancer remains difficult to treat, particularly in advanced stages, with treatment resistance contributing to cancer progression and mortality [3]. Radiation therapy represents a method used to treat locally advanced cancer and roughly half of cancer patients are treated with radiotherapy in conjunction with surgery and/or chemotherapy, as primary therapy [4]. Radiotherapy uses high doses of ionizing radiation (IR) to evoke cell death primarily by causing cellular DNA damage [5]. Resistance to radiation also known as radioresistance is a major clinical problem in various cancer types including glioblastoma [6], breast [7] as well as oesophageal adenocarcinoma [8]. Radioresistance can be defined as a process wherein the adaptation of tumour cells to radiotherapy-induced damage leads to developing resistance to IR. Radioresistance is a complex process involving alteration of several cellular mechanisms including DNA damage repair mechanisms, cell cycle arrest, oncogenes and tumour suppressor genes, tumour microenvironment changes, autophagy, cancer stem cells generation, tumour metabolism and altered regulation of reactive oxygen species [2,9]. In addition, cellular organelles are important targets of IR and they play a significant role in mediating radiation cytotoxic effects. It has been shown that radiation can damage the endoplasmic reticulum, induce changes in the ribosome, damage the lysosome, affect the biological properties and the signal transduction of the plasma membrane, and affect mitochondrial function [5]. DNA damage induced by IR can result in a massive alteration in mitochondria function as the mitochondrial DNA is genetically denser compared to the nuclear genome [10]. Mitochondria are doubled membraned organelles that play vital roles in physiological cellular processes such as energy production, macromolecule biosynthesis, gene expression, apoptosis, calcium homoeostasis and regulation of ROS production [11], [12], [13]. Many of these mitochondrial processes are altered in cancer [14] and increasing evidence suggests that altered mitochondria morphology and metabolism are associated with radioresistance [15]. In particular, energy metabolism reprogramming is an emerging hallmark of cancer [16] which plays a critical role in radioresponse [2]. Warburg effect is the best-known example of energy metabolism reprogramming in cancer, where cancer cells preferentially use the less efficient glycolytic pathway even in the presence of oxygen to generate energy and to produce important molecules sustaining cancer progression [17]. In recent years increasing evidence demonstrates that rewiring of mitochondrial function and metabolism plays an important role in tumour progression and response to treatment by modulating the mitochondrial energy production processes [15]. In this article have been reviewed the recent findings, summarised in Fig. 1 and in Table 1, describing the cellular and molecular mechanisms by which cancer rewires the function of the mitochondria and metabolism to enhance radioresistance, and the role that drugs targeting bioenergetic have in enhancing radiation response.

**Table 1:** Summary of the findings describing the mechanisms of radioresistance regulated by mitochondrial dysfunction and altered metabolism in cancer.

Altered cellular process	Authors	Cancer type	Mechanism of radioresistance	Ref
**Redox balance**	Lynam-Lennon, N. et al.	Oesophageal adenocarcinoma	Higher basal level of ROS	[15]
	Wei, Y. et al.	Non-small cell lung carcinoma	Lower radiation-induced ROS level	[19]
	Lynam-Lennon, N. et al.	Oesophageal adenocarcinoma	Maintenance of glutathione levels stable post irradiation	[21]
	Hanot, M. et al.	Tongue squamous cell carcinoma	Altered glutathione levels	[22]
	Chen, X. et al.	Oesophageal squamous cell carcinoma	Increased IDH2 expression	[24]
	Sun, J. et al.	CHO cells	MnSOD overexpression	[20]
	Hirose, K. et al.	Melanoma		[25]
	Kalen, A.L. et al.	Head-and-neck cancers		[26]
	Fisher, C.J. et al.	Pancreatic		[27]
**DNA repair**	Lynam-Lennon, N. et al.	Oesophageal adenocarcinoma	Increased repair of radiation-induced DNA damage	[21]
	Hyun, J. W. et al.	Leukaemia	Low OGG1 activity	[42]
	Ramdzan, Z.M. et al.	Breast, Lung, Glioblastoma, Colorectal	CUX1 overexpression	[46]
	Wang, Y. et al.	Non-small cell lung carcinoma	Downregulation of SSBP1	[54]
	Roy, K. et al.	Glioma	Methylation of the ATM promoter	[56]
	Squatrito, M. et al.	Glioblastoma	Loss of components in the ATM/Chk2/p53 pathway	[57]
	You, W.C. et al.	Glioblastoma	ATAD3A-mediated attenuation of DSB repairs	[58]
**Apoptosis**	He, Q. *et al*	Oesophageal squamous cell carcinoma	High LKB1 expression	[66]
	Wang, Z. et al.	Mouse embryonic fibroblasts	MKP1 inhibition of JNK-mediated apoptosis	[68]
	Candas, D. et al.	HER2-positive breast cancer	MKP1 overexpression	[148]
**Cell cycle checkpoints**	Wei, Y. et al.	Non-small cell lung carcinoma	Prolonged G2 arrest	[19]
	Cloos, *C. R.* et al.	Pancreatic	Supressed G2 activation increased cyclin B1 and CDK1	[77]
	Muschel, *R. J.,* et al.	Cervical cancer	Altered Cyclin B expression	[78]
	Liu, R. et al.	Colon, Glioblastoma, Breast	Increased SIRT3 activity	[80]
**Mitochondria-to-nucleus retrograde signaling**	Wei, Y. et al.	Non-small cell lung carcinoma	NF-κB/ PI3K/AKT2/mTOR activation	[19]
	Cloos, C. R. et al.	Pancreatic cancer	Mitochondrial DNA depletion	[77]
**Mitochondrial membrane potential**	Dong, Q. et al.	Oesophageal carcinoma	Modulation of BMI-1 expression	[84]
	Li, Y.L. et al.	Head and neck cancers	Increased GDF15 expression	[85]
**Mitochondrial ion channels**	Huang, L. et al.	Glioma	Overexpression of mtK_ATP_ channel	[86]
	Arif, T. et al.	Breast, lung Glioblastoma	VDAC1 overexpression	[14]
**Glycolysis**	Pitroda, S.P. et al.	Tongue squamous cell carcinoma	STAT1 regulation of energy metabolism	[91]
	De Schutter, H. et al.	Head and neck cancers	GLUT1 overexpression	[95]
	Kunkel, M. et al.	Oral cavity squamous carcinoma		[96]
	Blatt, S. et al.	Head and neck cancers	High levels of lactate	[97]
	Sandulache, V.C. et al.			[98]
	Hao, J. et al.	Prostate	Increased LDHA	[99]
**Glutamine metabolism**	Li, D. et al.	Pancreatic	Non-canonical glutamine metabolism pathway	[107]
	Xiang, L. et al.	Cervical	Increased GLS2 expression	[105]
**Oxidative phosphorylation**	Lynam-Lennon et al.	Oesophageal adenocarcinoma	Increased ATP5B expression	[15]
	Le Bleu, V.S. et al.	Breast	Increased PGC1α	[110]
	Viale, A. *et al*	Pancreatic	Increased expression of OXPHOS genes	[112]
	Grasso, D. et al.	Head and neck cancer	Increased OXPHOS metabolism	[113]

## Role of mitochondria in radioresistance

The latest findings describing the mitochondrial processes altered in cancer which are involved in radioresistance have been reviewed in this section.

### Mitochondria and redox balance in cancer

#### Ros and radioresistance

Mitochondria play a key role in the regulation of cellular redox balance thought modulation of Reactive Oxygen Species (ROS) generation [11]. Increased levels of ROS have been found in most cancers to support tumour development and growth; on the other side, tumour cells also have increased expression of antioxidants, which are responsible for ROS detoxification, suggesting that a delicate balance between ROS and antioxidants is required to sustain cancer cell growth [18]. However, IR induces abnormal production of ROS which, in turn, trigger DNA damage leading to cancer cell apoptosis. It has been shown that IR can cause mtDNA damage/deletion altering mitochondrial functions and leading to persistent production of cytotoxic superoxide [10]. Lynam-Lennon et al. have shown that radioresistant oesophageal adenocarcinoma cell line had significant higher basal levels of random mutations in the mitochondrial genome compared to its isogenic radiosentitive cell line, due to radiation bystander effects [15]. Mitochondria can adapt to DNA deletion damage by increasing their DNA copy number and quantity which can influence radiotherapy response [10,15]. In addition, ROS basal level was significantly higher in the radioresistant oesophageal adenocarcinoma cells, and an increase in ROS levels and in mitochondrial mass after IR was only detected in the radiosensitive cell. These findings suggested that the chronic oxidative stress present in the radioresistant cells inferred resistance to IR-induced oxidative stress [15]. Interestingly, it has been reported that mtDNA depletion induced significantly lower levels of ROS production after IR and a radioresistant phenotype compared to cells with functional mitochondria [19].

#### Antioxidants and radioresistance

Glutathione peroxidase and superoxide dismutase are ROS scavengers that function by catalysing ROS detoxification reactions to protect the cells against oxidative stress [20]. Increased levels of antioxidant enzymes have been linked to radioresistance in various cancer types. It has been showed that radioresistant oesophageal adenocarcinoma cells could maintain their basal level of glutathione post-irradiation stable, suggesting that this mechanism can play a role in their radioresistant phenotype [21]. *In vitro* models of head and neck cancer and Chinese hamster ovary cells showed that increased expression of antioxidants involved in the glutathione system, such as glutathione and glutathione peroxidase, was linked to radioresistance and increased survival [20,22]. However, in mouse squamous cell carcinoma derived cell line, glutathione and its associated enzymes played no part in their intrinsic radioresistance [23].

Isocitrate dehydrogenase 2 (IDH2) is a mitochondrial enzyme that catalyse the oxidative decarboxylation of isocitrate into alpha-ketoglutarate in the citric acid cycle. It has been shown that IDH2 played a key role in the radioresistance of oesophageal squamous cell carcinoma by increased the radiation-induced ROS level, oxidative damage, and cell apoptosis in IDH2 knockdown cells [24].

Manganese superoxide dismutase (MnSOD) is a mitochondrial antioxidant associated to radioresistance [25]. Several *in vitro* studies have shown that MnSOD overexpression is linked with increased cell survival and resistance to IR in various cancer types, suggesting that MnSOD might represent a potential therapeutic target [26,27]. MnSOD also plays a role in mitochondrial stability as its overexpression protected against the initial decrease in mitochondrial transmembrane potential (ΔΨ) after IR, an important step in the initiation of IR-induced apoptosis pathway [28].

Catalase is an antioxidant enzyme responsible for conversion of H_2_O_2,_ generated during cellular stress conditions, to water and oxygen. A study focused to determine whether increased catalase was radioprotective showed that mitochondrially targeted overexpression of the catalase transgene was having radioprotective effect *in vitro* and *in vivo,* suggesting a potential strategy for radioprotection of normal tissues during radiation therapy [28]. In addition, Epperly et al. [29,30] showed that overexpression of both mitochondrial MnSOD and mitochondrial catalase transgenes was superior to one alone for radioprotection of specific organs including *in vivo* models of oral cavity [30], bladder [31], lung [32] and oesophagus [33].

### Mitochondrial dysfunction, DNA repair and radioresistance

DNA repair mechanisms play a fundamental role in the control of response to IR, and enhanced DNA repair was associated with radioresistance in cancer [21]. A number of studies have shown that the mitochondrial DNA (mtDNA) is more sensitive to IR than nuclear DNA [34], [35], [36], possibly due to its close proximity to ROS and its denser genome [36,37]. Thymine glycol and 8-oxoGuanine are considered to be the major products of mtDNA oxidation damage induced by IR, contributing broadly to oxidative damage-induced mutations. DNA base alterations are repaired by base excision repair (BER) enzymes as demonstrated in a number of mammalian models [37], [38], [39]. Enzymes important in BER repair such as DNA glycosylases, abasic-endonucleases (APE), DNA polymerase, and DNA ligase have been found to be either localised to the mitochondria or actively transported to the mitochondria [40]. Oxoguanine glycosylase (OGG1) is a major DNA glycosylase enzyme involved in repair of 8‑hydroxy‑2′-deoxyguanosine mutation, and it has been shown to have an important role in mtDNA damage repair, a key mechanism regulating radiation response [37,41]. Hyun et al. showed that a loss of OGG1 was associated with enhanced radiosensitivity in acute lymphocytic leukaemia, thereby highlighting an important role of OGG1 in radioprotection [42]. In addition, the enzymatic activity of OGG1 is directly stimulated by both CUX1 [43,44] and CUX2 [45], haploinsufficient tumour suppressor genes which play an important role in radioresistance [46]. Interestingly, CUX1 gene copy number is increased in over 70% of human cancers, and its expression inversely correlates with patient survival [47,48]. CUX1 knockdown was lethal to cancer cells following ionizing radiation [46]. Several drugs that inhibit BER enzymes, such as PARP1 and APE1, are tested in the clinic with different treatment modalities (https://clinicaltrials.gov/) to treat cancer. However, BER enzymes exert essential functions in human tissues since over 30,000 base alterations/day are produced in healthy cells [49], thus its inhibition is associated with side effects. In contrast, CUX1 only functions as an auxiliary factor that accelerates repair of oxidative damage [44,50], therefore it is not essential in normal human cells [51] suggesting that CUX1 might represent an ideal therapeutic target for cancer treatment.

Mitochondrial Single Stranded DNA Binding Protein 1 (SSBP1) is part of the single-stranded DNA binding protein family which are guardians of genome stability controlling DNA damage response in the cell [52]. In an *in vitro* model of osteoblastoma it was shown that SSBP1 expression was linked to cancer progression, suggesting its use as a prognostic biomarker [53]. Downregulation of SSBP1 has been associated to increased radiosensitivity in lung cancer by decreasing the mtDNA copy number and mitochondrial morphology and function [54].

IR produces a wide variety of DNA damages. Double-strand breaks (DSBs) are considered to be the major effectors for IR-induced cell death [55]. Once DSBs occur, numerous sensor proteins,including ataxia-telangiectasia, Rad3-related, DNA-dependant protein kinase and ataxia-telangiectasia mutated, are recruited to the site of the damage (ATM) [4]. It has been shown that ATM plays an important role in glioblastoma response to IR, as cells lacking the ATM protein were highly sensitive to ionizing radiation and [56]. Another study showed that loss of components in the ATM/Chk2/p53 pathway was associated with radioresistance in a glioma mouse model, and it was demonstrated that Chk2 is required for glioma response to IR [57]. In addition, *in vitro* models of glioblastoma showed that mitochondrial enzyme ATAD3A plays a role in ATM function as silencing of ATAD3A was associated with attenuation of DSB repairs and enhanced radiosensitivity [58,59].

### Mitochondria, apoptosis and radioresistance

One of the hallmarks of cancer is the ability of cancer cells to avoid apoptosis [60]. Evasion of apoptosis in cancer is associated with treatment resistance to IR. IR primarily triggers the mitochondrial dependent pathway of apoptosis and therefore mitochondria dysfunction in cancer plays a crucial role in regulating treatment resistance and evasion of apoptosis [61]..

Human tumour suppressor LKB1 is a primary upstream kinase of adenosine monophosphate-activated protein kinase (AMPK) pathway, a necessary element in cell metabolism that is required for maintaining energy homoeostasis, metabolism, polarity, growth and autophagy [62], [63], [64]. Saigusa et al. showed that in locally advanced rectal cancer treated with neoadjuvant chemo-radiation therapy the expression of LKB1 was higher in patients with tumour recurrence and poor pathological response, demonstrating that LKB1 may play a role in treatment resistance [65]. In addition, it was demonstrated that overexpression of LKB1 induced radioresistance by inhibiting apoptosis and by activating autophagy, suggesting that LKB1 might be a novel therapeutic target to maximise radiation response in oesophageal cancer [66].

Mitogen-activated protein kinase (MAPK) phosphatase (MKP)−1 is the major member of MKPs that dephosphorylates and inactivates MAPK, a major signaling transduction molecules in apoptosis [67]. In a study was demonstrated that IR-induced MKP-1 played an anti-apoptotic function *via* inhibition of JNK-mediated proapoptotic pathway, and siRNA-mediated inhibition of MKP-1 significantly enhanced apoptotic response in irradiated cells [68].

### Mitochondria dysfunction and the cell cycle checkpoints in cancer cell radioresistance

Cell cycle checkpoints are often violated in cancer due to increased expression of growth factors and evading growth suppressors which lead to dysregulation of cell progression control mechanisms [16]. After exposure to IR, eukaryotic cells undergo a division delay which is reflected by increased time spent in the G2 portion of the cell cycle. The cell cycle checkpoints are regulated by cyclin-cyclin dependent kinases (CDK) complexes, and theG2/M checkpoint which controls progression to mitosis is primarily controlled by Cyclin B1-CDK1 complex activity [69]. Cyclin B1 has been found overexpressed in various cancers including oesophageal squamous cell carcinoma [70], non-small cell lung cancer [71], oral carcinoma [72], prostate adenocarcinoma [73], breast cancer [74], astrocytomas [75] and *Helicobacter pylori*-associated gastric MALT and MALT lymphoma [76]. It was shown that IR-induced G2 activation was suppressed by mtDNA depletion in human pancreas cancer cell and was accompanied by increased levels of Cyclin B1 and CDK1. The pancreatic cells with mtDNA depletion were more resistant to IR compared to cells with functional mitochondria [77]. Similarly, mtDNA depletion in lung cancer cells showed prolonged G2 arrest accompanied by a more radioresistant phenotype [19]. It has been shown that a cell division delay was induced when HeLa cells were irradiated in G2 phase which coincided with a lower level of cyclin B protein, identifying cyclin B as a key molecule in the cellular response to ionizing radiation [78].

SIRT3, a member of the sirtuin family of NAD(+)-dependent protein deacetylases in the mitochondria, regulates mitochondrial processes by promoting metabolic homoeostasis through regulation of mitochondrial protein deacetylation [79]. It has been demonstrated that SIRT3 expression was induced in a number of radiation-treated human cancer cells and xenografts, and it was showed that enhanced SIRT3 transcription and posttranslational modifications contributed to adaptive radioresistance in tumour [80].

### MtDNA depletion and radioresistance

Mitochondria-to-nucleus retrograde signalling (RTG) allows crosstalk between the mitochondria and nucleus influencing changes in a variety of cancer cellular phenotypes including survival, metastasis, drug resistance, stemness and metabolism [81]. RTG can be influenced by alterations in the copy number and mutations in mtDNA, respiratory chain component defects in the mitochondria, and loss of mitochondrial membrane potential [82]. Wei et al. showed that mtDNA depletion in lung cancer may induce radioresistance through activation of the RTG signalling pathway of NF-κB/PI3K/AKT2/mTOR [19]. This requires further study to determine if mtDNA alteration could be used as a predictor of response to radiation therapy and whether NF-κB/PI3K/AKT2/mTOR pathway might represents a potential therapeutic target to enhance radiosensitivity.

### Mitochondrial membrane and radioresistance

The mitochondrial membrane potential (ΔΨ) generated by proton pumps (Complexes I, III and IV) is an essential component in the process of energy storage during oxidative phosphorylation [83]. A study showed that histone deacetylase inhibitors (HDAC inhibitor), an emerging anti-cancer therapy, reverse the acquired radioresistance in an *in vitro* oesophageal cancer by modulating polycomb complex protein BMI-1 expression leading to enhanced G2/M arrest, decreased ΔΨ, increased production of ROS, and enhanced radiation induced apoptosis [84]. Growth differentiation factor 15 (GDF15) is a member of the TGF-β super-family that alters the ΔΨ inducing ROS suppression. It has been shown than GDF15 promoted cancer stemness, and inhibition of GDF15 sensitised cells to IR [85].

Mitochondrial ion channels are present on both the inner and outer mitochondrial membrane and play important roles in protein transport, ATP production and Ca^2+^ uptake. Mitochondrial ATP-sensitive potassium channel (mtK_ATP_) channel has been shown to have a key role in glioma radioresistance by regulating the ROS activation of EKR pathway. High expression of mtK_ATP_ in glioma tissue correlated to shorter survival time and a high malignancy grade, suggesting that use of mtK_ATP_ channel blockers in glioma treatment may improve patient outcomes [86]. Voltage-dependent anion channel 1 (VDAC1) is a class of membrane channel acting as a gatekeeper at the outer mitochondrial membrane. VDAC1 is involved in various mitochondrial processes and serves as a link between glycolysis and OXPHOS due to its interaction with hexokinase [87]. It has been shown that depletion of VDAC1 led to inhibition of tumour development and growth of cancer cells in both *in vitro* and *in vivo* models of breast, lung and glioblastoma, as well as and induction of metabolic rewiring reversing the oncogenic properties of cancer cells [14].

## Role of metabolism in radioresistance

The alteration of the cellular metabolic pathways in tumour cells, also known as metabolic reprogramming, is considered one of the hallmarks of cancer [88]. Altered activation of oncogenes, inactivation of tumour suppressor genes and changes in cellular signaling pathways are crucial factors regulating metabolic change in tumour [89,90]. Increasing number of evidences suggest that metabolic reprograming in cancer is one of the major factors contributing to radioresistance to IR [91].Mitochondria play a key role in this process. Mitochondria can rapidly adapt to the increasing energy requirements of the tumour by changing energy production processes in the cells, and radioresistance has been associated with changes in the mitochondrial energy metabolism profile, mitochondrial size, morphology and functions [15]. Carbohydrates are the preferred source of cellular energy in the cells participating in the process of the metabolism of glucose by glycolysis and OXPHOS [92]. Alterations in the glycolytic metabolism in cancer has been shown to contribute to radioresistance development [91]. A class of transporter proteins, GLUT family, is required by the cell for glucose metabolism in the cell [93]. Increasing evidence suggests that high glucose transporter‑1 (GLUT‑1) expression is associated with radioresistance in cancer. Abnormal GLUT-1 expression in cancer is regulated by various factors including hypoxia and altered MAPK and PI3K/AKT signalling pathways [94].Overexpression of GLUT1 was linked to radioresistance in both head and neck squamous cell carcinoma [95] and oral cavity squamous cell carcinoma [96] making it an attractive target for enhancing radiosensitivity. A study showed that GLUT1 inhibitors led to sensitization of radioresistant breast cancer cells to IR, suggesting a potential role of GLUT1 inhibitors as anti-cancer agents [94]. Lactic acid/lactate is a product of glycolysis and high levels of lactate was associated with increased tumour recurrence and poor survival in head and neck cancer patients, suggesting that lactate may have a value as a predictive biomarker of patients survival [97]. Lactate levels have beenassociated with hypoxia-induced radioresistance. A study showed that acute perturbations in tumour lactate levels acted as a surrogate marker of radiation response, suggesting that lactate might be a quantitative biomarker of acute genotoxic stress that could inform clinical decision making [98]. Lactate dehydrogenase (LDHA) is the main metabolic enzyme for lactate generation,a terminal product from glycolysis. It has been demonstrated that knockdown of LDHA could sensitize radioresistant prostate cancer cells to radiotherapy, and proteomic analysis revealed potential protein markers of radioresistance, concluding that targeting LDHA combined with radiotherapy could increase radiosensitivity in radioresistant prostate cancer [99].

In addition, NOS2, the inducible nitric oxide synthase of iNOS, has been identified as a key molecule upregulated in many aggressive cancer types. Nitric oxide has been demonstrated to act as a modulator of cellular bioenergetic processes and of mitochondrial functions, and therefore has been associated to the alteration of metabolic pathways in cancer [100,101]. Interestingly, iNOS/NOS2-generated nitric oxide has been described as a contributor to radiation resistance in different types of cancer [102,103].

### Glutamate metabolism and radioresistance

Glutamate plays key role linking carbohydrate and amino acid metabolism via the tricarboxylic acid (TCA) cycle in the cell [104]. Glutamate is synthesized from glutamine as part of the glutamate–glutamine cycle by the enzyme glutaminase. Xiang et al. showed that a mitochondrial isoform of glutaminase, GLS2, was associated with radioresistance in cervical cancer and its inhibition led to increased radiosensitivity, suggesting that the inhibition of GLS2 may be a potential treatment strategy for radioresistant cervical cancer [105]. A study showed that pancreatic ductal adenocarcinoma tumour growth is facilitated by a KRAS-mediated reprogramming of glutamate metabolism and suggesting that inhibition of glutamine metabolism might potentially synergize with therapies that increase intracellular reactive oxygen species, such as chemotherapy and radiation [106]. It has been shown that pancreatic cancer stem cells relied on the glutamine metabolism pathway and the inhibition of this pathway led to increased radiosensitivity by accumulation of ROS [107].

### OXPHOS and radioresistance

Rewiring of energy metabolism in cancer is most commonly associated with a switch from OXPHOS to glycolysis [108]. However increasing evidence demonstrates that cancer cells can use a wide range of energetic profiles, and OXPHOS represents a major source of energy production [109]. It has been previously demonstrated a role for mitochondrial dysfunction and metabolic reprogramming in radioresistance, showing that oesophageal adenocarcinoma radioresistant cells had altered bioenergetics with significantly increased intracellular ATP levels associated to enhanced mitochondrial respiration. Radioresistant cells also demonstrated metabolic plasticity, efficiently switching between the glycolysis and oxidative phosphorylation energy metabolism pathways, accompanied by enhanced clonogenic survival [15]. ATP5B expression, a marker of oxidative phosphorylation, was significantly increased in patients who had a poor pathological response to neoadjuvant CRT, showing a role for specific mitochondrial alterations and metabolic remodelling in the radioresistance of oesophageal adenocarcinoma [15]. A study showed that circulating cancer cells in a mice model of breast cancer had increased expression of peroxisome proliferator-activated receptor gamma coactivator 1-alpha (PGC1α). PGC1α overexpression was associated to a regulation of mitochondrial function by increasing mitochondrial biogenesis and by a preferential use of OXPHOS [110]. It has been also shown that the dependency of pancreatic cancer stem cells on OXPHOS is determined by the balance between PGC1α and Myc [111]. Viale et al. showed that surviving cells from oncogene ablation in pancreatic cancer were reliant on OXPHOS and responsible for tumour relapse, while inhibition of OXPHOS decreased mitochondrial respiration and the ability of the surviving cells to form spheres affecting their survival [112]. Acquired radioresistance was also associated with a shift from a glycolytic to a more oxidative metabolism and to an increased number of mitochondria with a higher mtDNA content [113].

Cancer rewires the function of the mitochondria and metabolism to enhance resistance to ionising radiation. Radioresistant phenotype has been associated to alteration of ROS generation, antioxidants level, DNA repair mechanisms, apoptosis, cell cycle checkpoints, mitochondria-to-nucleus retrograde signaling (RTG), membrane potential (ΔΨ), glycolysis, glutamine metabolism and oxidative phosphorylation (OXPHOS). VDAC1, voltage-dependent anion channel 1; TCA, tricarboxylic acid cycle; ETC, electron transport chain; ATP, adenosine triphosphate; GDF15, growth differentiation factor 15; BMI-1, polycomb complex protein BMI-1; ROS, reactive oxygen species; mtK_ATP_, mitochondrial ATP-sensitive potassium channel; PGC1α, Peroxisome proliferator-activated receptor gamma coactivator 1-alpha; Cdk1, cyclin-dependent kinase 1; NF-Kb, nuclear Factor kappa-light-chain-enhancer of activated B cells; PI3K, phosphoinositide 3-kinases; AKT2, RAC-beta serine/threonine-protein kinase; mTOR, mammalian target of rapamycin; MKP1, mitogen-activated protein kinase phosphatase 1; LKB1, serine/threonine-protein kinase STK11; JNK, c-Jun N-terminal kinase.

## Genetic regulation of mitochondria energy metabolism and radioresistance

A set of genes and transcription factors encoded by the nuclear genome regulate mitochondrional biogenesis, functions and trasnscriptional activity. Alteration of these genes are also associated to radioresistance through modulation of mitochondrial function. For example, PGC1α is a co-transcriptional regulation factor important in mitochondrial biogenesis, and upregulation of PGC1α has been linked to chemoresistance and altered energy metabolism in cancer stem cells [111,114]. PGC1α has been linked to radioresistance due to its role in the activation of many others transcription factors including nuclear respiratory factor 1 (NRF1) and 2 (NRF2) which, in turn, are linked to radiation response in cancer [115]. NRF-1 has been linked to the transcriptional control of genes involved in mitochondrial function and biogenesis and it was demonstrated that NRF1 was a direct target of miR-504 [116]. In an *in vitro* model of nasopharyngeal carcinoma, radioresistance was associated with upregulated expression of miR-504, leading to lower levels of NRF1. Serum from nasopharyngeal carcinoma patients showed that miR-504 was up-regulated during different weeks of radiotherapy and correlated with tumour volume, lymph nodes, and metastasis stages, demonstrating that miR-504 regulated radioresistance by down-regulating the expression of NRF1 [117]. NRF2 is a transcriptional regulator of cytoprotective enzyme encoding genes [116] and several studies have shown that aberrant NRF2 regulation is linked to both radioresistance and chemoresistance in various cancers such as lung [118], prostate [119], breast [120], ovarian [121] and oesophageal [122]. These evidences suggest that NRF2 targeting may be a potential treatment strategy for overcoming resistance to treatment. NRF-independent mitochondrial gene expression regulation is orchestrated by other factors such as Sp1 [123]. Sp1 is a transcription factor which regulates mitochondrial functions [124,125]. Deng et al. showed that high Sp1 expression levels were correlated to cervical cancer progression, and that knockdown of Sp1 significantly enhanced the cellular response to radiation by inducing G2/M arrest, suggesting that Sp1 might represent a potential therapeutic target in cervical cancer [126]. Tumour hypoxia is an important contributor to radioresistance. Hypoxic microenvironment is associated with modulation of expression of genes regulating tumour survival and growth [127]. Hypoxia-inducible factor-1 (HIF-1) is a protein that activates the transcription of many genes involved in angiogenesis, glucose metabolism, cell proliferation and invasion. HIF-1α is overexpressed in human cancer as a result of intratumoral hypoxia as well as genetic alterations [128]. For example, a study showed that radioresistance in glioblastoma is sustained by MEK/ERK regulation of HIF-1α. Signal transduction-based chemotherapy radiosensitised glioblastoma cancer cells by interfering with MEK/ERK pathway preventing HIF-1α-mediated hypoxic cell survival and radiation escape [129]. Interestingly, increasing evidence demonstrates that the switch from oxidative to glycolytic metabolism is an active response to hypoxia mediated by HIF-1α. HIF-1α gene expression alteration in cancer has been associated with altered mitochondrial mass and metabolism, along with increased glucose to pyruvate conversion, and increased cellular influx of glucose, positioning HIF1 as a lead target in radioresistance treatment [130], [131], [132]. In addition, a number of studies showed that hypoxia conferred resistance to irradiation through activation of autophagy [133], *via* c-Jun-mediated Beclin1 expression in lung cancer [134], and by the HIF-1α/miR-210/Bcl-2 pathway in colon cancer [135].

## Targeting mitochondrial energetics for enhanced radiosensitivity

Targeting mitochondrial energetics has emerged as a promising cancer treatment. Studies investigating the role that bioenergetic drugs play in radiation response are increasing [136]. Metformin is a member of the biguanide class of drugs and is widely used as an antidiabetic drug worldwide to treat Type II diabetes [114]. Interestingly, several studies have shown that metformin might have a role as antineoplastic agent, and that metformin is linked to enhanced radiation response in cancer. Storozhuk et al. demonstrated that metformin inhibited lung cancer cell growth and sensitised them to IR through ATM-AMPK signalling modulation [137]. In another study, metformin *in vitro* treatment of oesophageal cancer cells was shown to act as radiosensitiser leading to an increase in apoptosis, G0/G1 arrest and AMPK activation [138]. In addition, metformin was shown to lead to increased radiosensitivity in pancreatic cancer *in vitro,* and these effects were abrogated by AMPK inhibition [139]. Jin et al. showed that metformin treatment in colorectal cancer cells was linked to increased expression of AMPK and enhanced radioresistance, and AMPK inhibition led to increased radiosensitivity in these cells [140]. These evidences suggest that AMPK plays an important role in modulating metformin radiosensitiser effects. It has also been reported that metformin sensitised p53-deficient colorectal cancer cells to IR by reducing DNA repair protein levels, and by increasing the proportion of cells in G2/M phase [141]. In addition, it has been shown that resistance to metformin in cancer was linked to increased glycolysis and reduction in mitochondrial metabolism due to mutations in the electron transport chain or to enhanced hypoxia [111,142]. Benej et al. showed that papaverine, a FDA-approved vasodilator drug, acted as a mitochondria complex I inhibitor, and inhibition of this complex was directly responsible for increased oxygenation and enhanced radiation response in solid tumour. This study suggested that papaverine has the potential to become a clinical radiosensitisers with potentially few side effects as it did not sensitise well-oxygenated normal tissue to IR, thereby increasing the therapeutic index of radiotherapy [143]. In another study, it was demonstrated that inhibition of HIF-1α with 2-methoxyestradiol (2-MeOE2) significantly enhanced radiosensitivity in a radioresistant human melanoma cell model through targeting the glycolysis pathway [144]. Another study showed that dichloroacetate, a pyruvate dehydrogenase kinase (PDK) inhibitor currently being used to treat lactic acidosis, was able to alter glioblastoma cell metabolism by activating mitochondrial toward oxidative phosphorylation and by reversing the radiotherapy-induced glycolytic shift, demonstrating that dichloroacetate was able to sensitize glioblastoma cells to radiotherapy by modulating the metabolic activity of the tumour [145]. Finally, Buckley et al. showed that pyrazinib, a pyrazine phenol small molecule drug with anti-angiogenic and anti-metabolic activity, was shown to increase radiosensitivity in a model of radioresistant oesophageal adenocarcinoma by modulating mitochondrial bioenergetics, reducing measures of oxidative phosphorylation and glycolysis, therefore supporting further development of pyrazinib as a novel therapeutic radiosensitiser in oesophageal adenocarcinoma [146].

## Discussion

Ionising radiation (IR) is one of the most used therapeutic method for the treatment of many cancers. However, due to development of radioresistance by the tumour, IR remains primarily a conservative cancer treatment. Radioresistance is described as a complex process in which the cancerous tissue adapts to the cellular changes induced by IR and develops resistance by modulating multiple genes, factors, and cellular processes. Such processes include alteration of DNA damage repair mechanism, cell cycle, oncogenes, tumour suppressor genes, autophagy, cellular metabolism, and reactive oxygen species. These altered processes are regulated by mitochondria and implicated in cancer treatment response Increasing evidence suggests that mitochondria dysfunction and altered metabolism in cancer plays a key role in radioresistance. In fact, radioresistance in cancer has been associated with changes in the mitochondrial energy metabolism profile, mitochondrial size and number, mitochondrial morphology and function accompanied by increased mitochondrial mutation rate, respiration and intracellular ATP levels [15]. In addition, crosstalk between the mitochondria and nucleus is required for normal cellular function, therefore dysfunction in the mitochondria can lead to alterations in nuclear gene expression and *vice versa* [81]. A number of studies have shown that alteration of nuclear encoded genes and transcription factors in cancer, which regulate mitochondria processes, can lead to mitochondrial dysfunction and to radioresistance [15,^112^,147]. In this review we detailed the mechanisms by which cancer rewires the function of the mitochondria and cellular metabolism to enhance resistance to IR. Fig. 1 shows the various processes and mechanisms described in this article by which the alterations in mitochondrial function and metabolism play a role in radioresistance. Importantly, there are no available radiosensitisers on the market that can be used to increase response in radioresistant cancer, thus, there is increasing interest in identifying the mechanisms regulating radioresistance in order to development targeted novel treatments to enhance radiosensitivity. In this scenario, a number of studies showed that drugs targeting mitochondria bioenergetics such as metformin [137], [138], [139], [140], [141] and pyrazinib [146] might have some promise as radiosensitisers. Research focused on development of therapeutics targeting radioresistance through rewiring of mitochondria dysfunctions and altered metabolism in cancer will translate to the development of novel radiosensitisers, with the ultimate goal of improving response to radiotherapy in cancer patients.

## CRediT authorship contribution statement

**Emma McCann:** Investigation, Writing - original draft. **Jacintha O'Sullivan:** Conceptualization, Writing - review & editing. **Simone Marcone:** Conceptualization, Writing - review & editing.

## Declaration of Competing Interest

The authors declare no competing interests.

## References

[1] Shridhar R., Almhanna K., Meredith K.L., Biagioli M.C., Chuong M.D., Cruz A., Hoffe S.E. (2013). Radiation therapy and esophageal cancer. Cancer Control.

[2] Tang L., Wei F., Wu Y., He Y., Shi L., Xiong F., Gong Z., Guo C., Li X., Deng H., Cao K., Zhou M., Xiang B., Li Y., Li G., Xiong W., Zeng Z. (2018). Role of metabolism in cancer cell radioresistance and radiosensitization methods. J. Exp. Clin. Cancer Res..

[3] Seyfried T.N., Huysentruyt L.C. (2013). On the origin of cancer metastasis. Crit. Rev. Oncog..

[4] Kim W., Lee S., Seo D., Kim D., Kim K., Kim E., Kang J., Seong K.M., Youn H., Youn B. (2019). Cellular Stress Responses in Radiotherapy. Cells.

[5] Wang J.S., Wang H.J., Qian H.L. (2018). Biological effects of radiation on cancer cells. Mil. Med. Res..

[6] Ahmed K.A., Chinnaiyan P., Fulp W.J., Eschrich S., Torres-Roca J.F., Caudell J.J. (2015). The radiosensitivity index predicts for overall survival in glioblastoma. Oncotarget.

[7] Jameel J.K., Rao V.S., Cawkwell L., Drew P.J. (2004). Radioresistance in carcinoma of the breast. Breast.

[8] Walsh T.N., Noonan N., Hollywood D., Kelly A., Keeling N., Hennessy T.P. (1996). A comparison of multimodal therapy and surgery for esophageal adenocarcinoma. New Engl. J. Med..

[9] Diehn M., Cho R.W., Lobo N.A., Kalisky T., Dorie M.J., Kulp A.N., Qian D., Lam J.S., Ailles L.E., Wong M., Joshua B., Kaplan M.J., Wapnir I., Dirbas F.M., Somlo G., Garberoglio C., Paz B., Shen J., Lau S.K., Quake S.R., Brown J.M., Weissman I.L., Clarke M.F. (2009). Association of reactive oxygen species levels and radioresistance in cancer stem cells. Nature.

[10] Kam W.W., Banati R.B. (2013). Effects of ionizing radiation on mitochondria. Free Radic. Biol. Med..

[11] Wallace D.C. (2012). Mitochondria and cancer. Nat. Rev. Cancer.

[12] Vakifahmetoglu-Norberg H., Ouchida A.T., Norberg E. (2017). The role of mitochondria in metabolism and cell death. Biochem. Biophys. Res. Commun..

[13] J.M. Berg, J.L. Tymoczko, L. Stryer, Biochemistry, W.H.Freeman, New York, 2007.

[14] Arif T., Paul A., Krelin Y., Shteinfer-Kuzmine A., Shoshan-Barmatz V. (2018). Mitochondrial VDAC1 Silencing Leads to Metabolic Rewiring and the Reprogramming of Tumour Cells into Advanced Differentiated States. Cancers (Basel).

[15] Lynam-Lennon N., Maher S.G., Maguire A., Phelan J., Muldoon C., Reynolds J.V., O'Sullivan J. (2014). Altered mitochondrial function and energy metabolism is associated with a radioresistant phenotype in oesophageal adenocarcinoma. PLoS ONE.

[16] Hanahan D., Weinberg R.A. (2011). Hallmarks of cancer: the next generation. Cell.

[17] Warburg O.H., Dickens F., Kaiser-Wilhelm-Institut für B. (1930). The Metabolism of tumours; Investigations from the Kaiser Wilhelm Institute For biology, Berlin-Dahlem.

[18] Liou G.Y., Storz P. (2010). Reactive oxygen species in cancer. Free Radic Res.

[19] Wei Y., Chen L., Xu H., Xie C., Zhou Y., Zhou F. (2018). Mitochondrial dysfunctions regulated radioresistance through mitochondria-to-nucleus retrograde signaling pathway of NF-κB/PI3K/AKT2/mTOR. Radiat. Res..

[20] Sun J., Chen Y., Li M., Ge Z. (1998). Role of antioxidant enzymes on ionizing radiation resistance. Free Radic. Biol. Med..

[21] Lynam-Lennon N., Reynolds J.V., Pidgeon G.P., Lysaght J., Marignol L., Maher S.G. (2010). Alterations in DNA repair efficiency are involved in the radioresistance of esophageal adenocarcinoma. Radiat. Res..

[22] Hanot M., Boivin A., Malésys C., Beuve M., Colliaux A., Foray N., Douki T., Ardail D., Rodriguez-Lafrasse C. (2012). Glutathione depletion and carbon ion radiation potentiate clustered DNA lesions, cell death and prevent chromosomal changes in cancer cells progeny. PLoS ONE.

[23] Miura M., Sasaki T. (1991). Role of glutathione in the intrinsic radioresistance of cell lines from a mouse squamous cell carcinoma. Radiat. Res..

[24] Chen X., Zhuo S., Xu W., Chen X., Huang D., Sun X., Cheng Y. (2020). Isocitrate dehydrogenase 2 contributes to radiation resistance of oesophageal squamous cell carcinoma via regulating mitochondrial function and ROS/pAKT signalling. Br. J. Cancer.

[25] Hirose K., Longo D.L., Oppenheim J.J., Matsushima K. (1993). Overexpression of mitochondrial manganese superoxide dismutase promotes the survival of tumor cells exposed to interleukin-1, tumor necrosis factor, selected anticancer drugs, and ionizing radiation. FASEB J..

[26] Kalen A.L., Sarsour E.H., Venkataraman S., Goswami P.C. (2006). Mn-superoxide dismutase overexpression enhances G2 accumulation and radioresistance in human oral squamous carcinoma cells. Antioxid. Redox Signal.

[27] Fisher C.J., Goswami P.C. (2008). Mitochondria-targeted antioxidant enzyme activity regulates radioresistance in human pancreatic cancer cells. Cancer Biol. Ther..

[28] Epperly M.W., Melendez J.A., Zhang X., Nie S., Pearce L., Peterson J., Franicola D., Dixon T., Greenberger B.A., Komanduri P., Wang H., Greenberger J.S. (2009). Mitochondrial targeting of a catalase transgene product by plasmid liposomes increases radioresistance *in vitro* and *in vivo*. Radiat. Res..

[29] Epperly M.W., Melendez J., Zhang X., Franicola D., Smith T., Greenberger J.S. (2007). Radioresistance induced by MnSOD overexpression in 32DCl 3murine hematopoietic progenitor cells is further increased by localization of a catalase transgene product to the mitochondria. Int. J. Radiat. Oncol. Biol. Phys..

[30] Epperly M.W., Wegner R., Kanai A.J., Kagan V., Greenberger E.E., Nie S., Greenberger J.S. (2007). Effects of MnSOD-plasmid liposome gene therapy on antioxidant levels in irradiated murine oral cavity orthotopic tumors. Radiat. Res..

[31] Kanai A.J., Zeidel M.L., Lavelle J.P., Greenberger J.S., Birder L.A., de Groat W.C., Apodaca G.L., Meyers S.A., Ramage R., Epperly M.W (2002). Manganese superoxide dismutase gene therapy protects against irradiation-induced cystitis. Am. J. Physiol. Renal Physiol..

[32] Carpenter M., Epperly M.W., Agarwal A., Nie S., Hricisak L., Niu Y., Greenberger J.S. (2005). Inhalation delivery of manganese superoxide dismutase-plasmid/liposomes protects the murine lung from irradiation damage. Gene. Ther..

[33] Stickle R.L., Epperly M.W., Klein E., Bray J.A., Greenberger J.S. (1999). Prevention of irradiation-induced esophagitis by plasmid/liposome delivery of the human manganese superoxide dismutase transgene. Radiat. Oncol. Investig..

[34] Niranjan B.G., Bhat N.K., Avadhani N.G. (1982). Preferential attack of mitochondrial DNA by aflatoxin B1 during hepatocarcinogenesis. Science.

[35] Backer J.M., Weinstein I.B. (1980). Mitochondrial DNA is a major cellular target for a dihydrodiol-epoxide derivative of benzo[*a*]pyrene. Science.

[36] LeDoux S.P., Driggers W.J., Hollensworth B.S., Wilson G.L. (1999). Repair of alkylation and oxidative damage in mitochondrial DNA. Mutat. Res..

[37] Bohr V.A., Stevnsner T., de Souza-Pinto N.C. (2002). Mitochondrial DNA repair of oxidative damage in mammalian cells. Gene.

[38] Pinz K.G., Bogenhagen D.F. (1998). Efficient repair of abasic sites in DNA by mitochondrial enzymes. Mol. Cell Biol..

[39] LeDoux S.P., Wilson G.L. (2001). Base excision repair of mitochondrial DNA damage in mammalian cells. Prog. Nucleic Acid Res. Mol. Biol..

[40] Zinovkina L.A. (2018). Mechanisms of Mitochondrial DNA Repair in Mammals. Biochemistry (Mosc).

[41] Wong R.S.Y. (2011). Apoptosis in cancer: from pathogenesis to treatment. J. Exp. Clin. Cancer Res. CR.

[42] Hyun J.W., Cheon G.J., Kim H.S., Lee Y.S., Choi E.Y., Yoon B.H., Kim J.S., Chung M.H. (2002). Radiation sensitivity depends on OGG1 activity status in human leukemia cell lines. Free Radic. Biol. Med..

[43] Ramdzan Z.M., Pal R., Kaur S., Leduy L., Bérubé G., Davoudi S., Vadnais C., Nepveu A. (2015). The function of CUX1 in oxidative DNA damage repair is needed to prevent premature senescence of mouse embryo fibroblasts. Oncotarget.

[44] Ramdzan Z.M., Vadnais C., Pal R., Vandal G., Cadieux C., Leduy L., Davoudi S., Hulea L., Yao L., Karnezis A.N., Paquet M., Dankort D., Nepveu A. (2014). RAS transformation requires CUX1-dependent repair of oxidative DNA damage. PLoS Biol..

[45] Pal R., Ramdzan Z.M., Kaur S., Duquette P.M., Marcotte R., Leduy L., Davoudi S., Lamarche-Vane N., Iulianella A., Nepveu A. (2015). CUX2 protein functions as an accessory factor in the repair of oxidative DNA damage. J. Biol. Chem..

[46] Ramdzan Z.M., Ginjala V., Pinder J.B., Chung D., Donovan C.M., Kaur S., Leduy L., Dellaire G., Ganesan S., Nepveu A. (2017). The DNA repair function of CUX1 contributes to radioresistance. Oncotarget.

[47] Ripka S., Neesse A., Riedel J., Bug E., Aigner A., Poulsom R., Fulda S., Neoptolemos J., Greenhalf W., Barth P., Gress T.M., Michl P. (2010). CUX1: target of Akt signalling and mediator of resistance to apoptosis in pancreatic cancer. Gut.

[48] Cancer Genome Atlas N. (2012). Comprehensive molecular characterization of human colon and rectal cancer. Nature.

[49] Lindahl T., Barnes D.E. (2000). Repair of endogenous DNA damage. Cold Spring Harb. Symp. Quant. Biol..

[50] Ramdzan Z.M., Pal R., Kaur S., Leduy L., Berube G., Davoudi S., Vadnais C., Nepveu A. (2015). The function of CUX1 in oxidative DNA damage repair is needed to prevent premature senescence of mouse embryo fibroblasts. Oncotarget.

[51] Wang T., Birsoy K., Hughes N.W., Krupczak K.M., Post Y., Wei J.J., Lander E.S., Sabatini D.M. (2015). Identification and characterization of essential genes in the human genome. Science.

[52] Wu Y., Lu J., Kang T. (2016). Human single-stranded DNA binding proteins: guardians of genome stability. Acta Biochim. Biophys. Sin. (Shanghai.

[53] Shapovalov Y., Hoffman D., Zuch D., de Mesy Bentley K.L., Eliseev R.A. (2011). Mitochondrial dysfunction in cancer cells due to aberrant mitochondrial replication. J. Biol. Chem..

[54] Wang Y., Hu L., Zhang X., Zhao H., Xu H., Wei Y., Jiang H., Xie C., Zhou Y., Zhou F. (2017). Downregulation of mitochondrial single stranded DNA binding protein (SSBP1) induces mitochondrial dysfunction and increases the radiosensitivity in non-small cell lung cancer cells. J. Cancer.

[55] Vignard J., Mirey G., Salles B. (2013). Ionizing-radiation induced DNA double-strand breaks: a direct and indirect lighting up. Radiother. Oncol..

[56] Roy K., Wang L., Makrigiorgos G.M., Price B.D. (2006). Methylation of the ATM promoter in glioma cells alters ionizing radiation sensitivity. Biochem. Biophys. Res. Commun..

[57] Squatrito M., Brennan C.W., Helmy K., Huse J.T., Petrini J.H., Holland E.C. (2010). Loss of ATM/Chk2/p53 pathway components accelerates tumor development and contributes to radiation resistance in gliomas. Cancer Cell.

[58] You W.C., Chiou S.H., Huang C.Y., Chiang S.F., Yang C.L., Sudhakar J.N., Lin T.Y., Chiang I.P., Shen C.C., Cheng W.Y., Lin J.C., Shieh S.H., Chow K.C. (2013). Mitochondrial protein ATPase family, AAA domain containing 3A correlates with radioresistance in glioblastoma. Neuro Oncol..

[59] S. De Vleeschouwer, Glioblastoma, 2017.

[60] Fouad Y.A., Aanei C. (2017). Revisiting the hallmarks of cancer. Am. J. Cancer Res..

[61] Cao X., Wen P., Fu Y., Gao Y., Qi X., Chen B., Tao Y., Wu L., Xu A., Lu H., Zhao G. (2019). Radiation induces apoptosis primarily through the intrinsic pathway in mammalian cells. Cell Signal.

[62] Momcilovic M., Shackelford D.B. (2015). Targeting LKB1 in cancer - exposing and exploiting vulnerabilities. Br. J. Cancer.

[63] Gan R.Y., Li H.B. (2014). Recent progress on liver kinase B1 (LKB1): expression, regulation, downstream signaling and cancer suppressive function. Int. J. Mol. Sci..

[64] Shackelford D.B., Shaw R.J. (2009). The LKB1-AMPK pathway: metabolism and growth control in tumour suppression, Nature reviews. Cancer.

[65] Saigusa S., Inoue Y., Tanaka K., Toiyama Y., Kawamura M., Okugawa Y., Okigami M., Hiro J., Uchida K., Mohri Y., Kusunoki M. (2013). Significant correlation between LKB1 and LGR5 gene expression and the association with poor recurrence-free survival in rectal cancer after preoperative chemoradiotherapy. J. Cancer Res. Clin. Oncol..

[66] He Q., Li J., Dong F., Cai C., Zou X. (2017). LKB1 promotes radioresistance in esophageal cancer cells exposed to radiation, by suppression of apoptosis and activation of autophagy via the AMPK pathway. Mol. Med. Rep..

[67] Johnson G.L., Lapadat R. (2002). Mitogen-activated protein kinase pathways mediated by ERK, JNK, and p38 protein kinases. Science.

[68] Wang Z., Cao N., Nantajit D., Fan M., Liu Y., Li J.J. (2008). Mitogen-activated protein kinase phosphatase-1 represses c-Jun NH2-terminal kinase-mediated apoptosis via NF-kappaB regulation. J. Biol. Chem..

[69] Pezzella Francesco, Tavassoli Mahvash, Kerr David J (2019). Oxford Textbook of Cancer Biology.

[70] Murakami H., Furihata M., Ohtsuki Y., Ogoshi S. (1999). Determination of the prognostic significance of cyclin B1 overexpression in patients with esophageal squamous cell carcinoma. Virchows Archiv.

[71] Soria J.C., Jang S.J., Khuri F.R., Hassan K., Liu D., Hong W.K., Mao L. (2000). Overexpression of cyclin B1 in early-stage non-small cell lung cancer and its clinical implication. Cancer Res..

[72] Kushner J., Bradley G., Young B., Jordan R.C.K. (1999). Aberrant expression of cyclin A and cyclin B1 proteins in oral carcinoma. J. Oral Pathol. Med..

[73] Kallakury B.V.S., Sheehan C.E., Rhee S.J., Fisher H.A.G., Kaufman R.P., Rifkin M.D., Ross J.S. (1999). The prognostic significance of proliferation-associated nucleolar protein p120 expression in prostate adenocarcinoma. Cancer.

[74] Kawamoto H., Koizumi H., Uchikoshi T. (1997). Expression of the G2-M checkpoint regulators cyclin B1 and cdc2 in nonmalignant and malignant human breast lesions: immunocytochemical and quantitative image analyses. Am. J. Pathol..

[75] Allan K., Jordan R.C., Ang L.C., Taylor M., Young B. (2000). Overexpression of cyclin A and cyclin B1 proteins in astrocytomas. Arch. Pathol. Lab. Med..

[76] Banerjee S.K., Weston A.P., Zoubine M.N., Campbell D.R., Cherian R. (2000). Expression of Cdc2 and Cyclin B1 in Helicobacter pylori-Associated Gastric MALT and MALT Lymphoma: relationship to Cell Death, Proliferation, and Transformation. Am. J. Pathol..

[77] Cloos C.R., Daniels D.H., Kalen A., Matthews K., Du J., Goswami P.C., Cullen J.J. (2009). Mitochondrial DNA depletion induces radioresistance by suppressing G2 checkpoint activation in human pancreatic cancer cells. Radiat. Res..

[78] Muschel R.J., Zhang H.B., Iliakis G., McKenna W.G. (1991). Cyclin B expression in HeLa cells during the G2 block induced by ionizing radiation. Cancer Res.

[79] Lombard D.B., Tishkoff D.X., Bao J. (2011). Mitochondrial sirtuins in the regulation of mitochondrial activity and metabolic adaptation. Handb. Exp. Pharmacol..

[80] Liu R., Fan M., Candas D., Qin L., Zhang X., Eldridge A., Zou J.X., Zhang T., Juma S., Jin C., Li R.F., Perks J., Sun L.Q., Vaughan A.T., Hai C.X., Gius D.R., Li J.J. (2015). CDK1-Mediated SIRT3 activation enhances mitochondrial function and tumor radioresistance. Mol. Cancer Ther..

[81] Yang D., Kim J. (2019). Mitochondrial retrograde signalling and metabolic alterations in the tumour microenvironment. Cells.

[82] Guha M., Avadhani N.G. (2013). Mitochondrial retrograde signaling at the crossroads of tumor bioenergetics, genetics and epigenetics. Mitochondrion.

[83] Zorova L.D., Popkov V.A., Plotnikov E.Y., Silachev D.N., Pevzner I.B., Jankauskas S.S., Babenko V.A., Zorov S.D., Balakireva A.V., Juhaszova M., Sollott S.J., Zorov D.B. (2018). Mitochondrial membrane potential. Anal. Biochem..

[84] Dong Q., Sharma S., Liu H., Chen L., Gu B., Sun X., Wang G. (2014). HDAC inhibitors reverse acquired radio resistance of KYSE-150R esophageal carcinoma cells by modulating Bmi-1 expression. Toxicol. Lett..

[85] Li Y.L., Chang J.T., Lee L.Y., Fan K.H., Lu Y.C., Li Y.C., Chiang C.H., You G.R., Chen H.Y., Cheng A.J. (2017). GDF15 contributes to radioresistance and cancer stemness of head and neck cancer by regulating cellular reactive oxygen species via a SMAD-associated signaling pathway. Oncotarget.

[86] Huang L., Li B., Tang S., Guo H., Li W., Huang X., Yan W., Zou F. (2015). Mitochondrial KATP Channels Control Glioma Radioresistance by Regulating ROS-Induced ERK Activation. Mol. Neurobiol..

[87] Shoshan-Barmatz V., Ben-Hail D., Admoni L., Krelin Y., Tripathi S.S. (2015). The mitochondrial voltage-dependent anion channel 1 in tumor cells. Biochimica et Biophysica Acta (BBA) - Biomembranes.

[88] Yoshida G.J. (2015). Metabolic reprogramming: the emerging concept and associated therapeutic strategies. J. Exp. Clin. Cancer Res..

[89] Priolo C., Pyne S., Rose J., Regan E.R., Zadra G., Photopoulos C., Cacciatore S., Schultz D., Scaglia N., McDunn J., De Marzo A.M., Loda M. (2014). AKT1 and MYC induce distinctive metabolic fingerprints in human prostate cancer. Cancer Res..

[90] Yang L., Hou Y., Yuan J., Tang S., Zhang H., Zhu Q., Du Y.E., Zhou M., Wen S., Xu L., Tang X., Cui X., Liu M. (2015). Twist promotes reprogramming of glucose metabolism in breast cancer cells through PI3K/AKT and p53 signaling pathways. Oncotarget.

[91] Pitroda S.P., Wakim B.T., Sood R.F., Beveridge M.G., Beckett M.A., MacDermed D.M., Weichselbaum R.R., Khodarev N.N. (2009). STAT1-dependent expression of energy metabolic pathways links tumour growth and radioresistance to the Warburg effect. BMC Med..

[92] Li Z., Zhang H. (2016). Reprogramming of glucose, fatty acid and amino acid metabolism for cancer progression. Cell Mol. Life Sci..

[93] Mueckler M., Thorens B. (2013). The SLC2 (GLUT) family of membrane transporters. Mol. Aspects Med..

[94] Fang J., Zhou S.H., Fan J., Yan S.X. (2015). Roles of glucose transporter-1 and the phosphatidylinositol 3-kinase/protein kinase B pathway in cancer radioresistance (review). Mol. Med. Rep..

[95] De Schutter H., Landuyt W., Verbeken E., Goethals L., Hermans R., Nuyts S. (2005). The prognostic value of the hypoxia markers CA IX and GLUT 1 and the cytokines VEGF and IL 6 in head and neck squamous cell carcinoma treated by radiotherapy +/- chemotherapy. BMC Cancer.

[96] Kunkel M., Moergel M., Stockinger M., Jeong J.H., Fritz G., Lehr H.A., Whiteside T.L. (2007). Overexpression of GLUT-1 is associated with resistance to radiotherapy and adverse prognosis in squamous cell carcinoma of the oral cavity. Oral Oncol..

[97] Blatt S., Voelxen N., Sagheb K., Pabst A.M., Walenta S., Schroeder T., Mueller-Klieser W., Ziebart T. (2016). Lactate as a predictive marker for tumor recurrence in patients with head and neck squamous cell carcinoma (HNSCC) post radiation: a prospective study over 15 years. Clin. Oral Investig..

[98] Sandulache V.C., Chen Y., Skinner H.D., Lu T., Feng L., Court L.E., Myers J.N., Meyn R.E., Fuller C.D., Bankson J.A., Lai S.Y. (2015). Acute tumor lactate perturbations as a biomarker of genotoxic stress: development of a biochemical model. Mol. Cancer Ther..

[99] Hao J., Graham P., Chang L., Ni J., Wasinger V., Beretov J., Deng J., Duan W., Bucci J., Malouf D., Gillatt D., Li Y. (2016). Proteomic identification of the lactate dehydrogenase A in a radioresistant prostate cancer xenograft mouse model for improving radiotherapy. Oncotarget.

[100] Ridnour L.A., Thomas D.D., Switzer C., Flores-Santana W., Isenberg J.S., Ambs S., Roberts D.D., Wink D.A. (2008). Molecular mechanisms for discrete nitric oxide levels in cancer. Nitric Oxide.

[101] Glynn S.A., Boersma B.J., Dorsey T.H., Yi M., Yfantis H.G., Ridnour L.A., Martin D.N., Switzer C.H., Hudson R.S., Wink D.A., Lee D.H., Stephens R.M., Ambs S. (2010). Increased NOS2 predicts poor survival in estrogen receptor-negative breast cancer patients. J. Clin. Invest..

[102] Matsumoto H., Hayashi S., Hatashita M., Ohnishi K., Shioura H., Ohtsubo T., Kitai R., Ohnishi T., Kano E. (2001). Induction of radioresistance by a nitric oxide-mediated bystander effect. Radiat. Res..

[103] Cardnell R.J., Mikkelsen R.B. (2011). Nitric oxide synthase inhibition enhances the antitumor effect of radiation in the treatment of squamous carcinoma xenografts. PLoS ONE.

[104] Walker M.C., van der Donk W.A. (2016). The many roles of glutamate in metabolism. J. Ind. Microbiol. Biotechnol..

[105] Xiang L., Xie G., Liu C., Zhou J., Chen J., Yu S., Li J., Pang X., Shi H., Liang H. (2013). Knock-down of glutaminase 2 expression decreases glutathione, NADH, and sensitizes cervical cancer to ionizing radiation. Biochim. Biophys. Acta.

[106] Son J., Lyssiotis C.A., Ying H., Wang X., Hua S., Ligorio M., Perera R.M., Ferrone C.R., Mullarky E., Shyh-Chang N., Kang Y., Fleming J.B., Bardeesy N., Asara J.M., Haigis M.C., DePinho R.A., Cantley L.C., Kimmelman A.C. (2013). Glutamine supports pancreatic cancer growth through a KRAS-regulated metabolic pathway. Nature.

[107] Li D., Fu Z., Chen R., Zhao X., Zhou Y., Zeng B., Yu M., Zhou Q., Lin Q., Gao W., Ye H., Zhou J., Li Z., Liu Y. (2015). Inhibition of glutamine metabolism counteracts pancreatic cancer stem cell features and sensitizes cells to radiotherapy. Oncotarget.

[108] DeBerardinis R.J., Chandel N.S. (2016). Fundamentals of cancer metabolism. Sci. Adv..

[109] Porporato P.E., Filigheddu N., Pedro J.M.B., Kroemer G., Galluzzi L. (2018). Mitochondrial metabolism and cancer. Cell Res..

[110] LeBleu V.S., O'Connell J.T., Gonzalez Herrera K.N., Wikman H., Pantel K., Haigis M.C., de Carvalho F.M., Damascena A., Domingos Chinen L.T., Rocha R.M., Asara J.M., Kalluri R. (2014). PGC-1α mediates mitochondrial biogenesis and oxidative phosphorylation in cancer cells to promote metastasis. Nat. Cell Biol..

[111] Sancho P., Burgos-Ramos E., Tavera A., Bou Kheir T., Jagust P., Schoenhals M., Barneda D., Sellers K., Campos-Olivas R., GraAaAaAeA∼a O., Viera C.R., Yuneva M., Sainz B., Heeschen C. (2015). MYC/PGC-1[alpha] balance determines the metabolic phenotype and plasticity of pancreatic cancer stem cells. Cell Metab..

[112] Viale A., Pettazzoni P., Lyssiotis C.A., Ying H., Sánchez N., Marchesini M., Carugo A., Green T., Seth S., Giuliani V., Kost-Alimova M., Muller F., Colla S., Nezi L., Genovese G., Deem A.K., Kapoor A., Yao W., Brunetto E., Kang Y., Yuan M., Asara J.M., Wang Y.A., Heffernan T.P., Kimmelman A.C., Wang H., Fleming J.B., Cantley L.C., DePinho R.A., Draetta G.F. (2014). Oncogene ablation-resistant pancreatic cancer cells depend on mitochondrial function. Nature.

[113] Grasso D., Medeiros H.C.D., Zampieri L.X., Bol V., Danhier P., van Gisbergen M.W., Bouzin C., Brusa D., Gregoire V., Smeets H., Stassen A.P.M., Dubois L.J., Lambin P., Dutreix M., Sonveaux P. (2020). Fitter Mitochondria Are Associated With Radioresistance in Human Head and Neck SQD9 Cancer Cells. Front. Pharmacol..

[114] Bokil A., Sancho P. (2019). Mitochondrial determinants of chemoresistance. Cancer Drug Resist..

[115] Jornayvaz F.R., Shulman G.I. (2010). Regulation of mitochondrial biogenesis. Essays Biochem..

[116] Zhou S., Ye W., Shao Q., Zhang M., Liang J. (2013). Nrf2 is a potential therapeutic target in radioresistance in human cancer. Crit. Rev. Oncol. Hematol..

[117] Zhao L., Tang M., Hu Z., Yan B., Pi W., Li Z., Zhang J., Zhang L., Jiang W., Li G., Qiu Y., Hu F., Liu F., Lu J., Chen X., Xiao L., Xu Z., Tao Y., Yang L., Bode A.M., Dong Z., Zhou J., Fan J., Sun L., Cao Y. (2015). miR-504 mediated down-regulation of nuclear respiratory factor 1 leads to radio-resistance in nasopharyngeal carcinoma. Oncotarget.

[118] Singh A., Misra V., Thimmulappa R.K., Lee H., Ames S., Hoque M.O., Herman J.G., Baylin S.B., Sidransky D., Gabrielson E., Brock M.V., Biswal S. (2006). Dysfunctional KEAP1-NRF2 interaction in non-small-cell lung cancer. PLoS Med..

[119] Zhang P., Singh A., Yegnasubramanian S., Esopi D., Kombairaju P., Bodas M., Wu H., Bova S.G., Biswal S. (2010). Loss of Kelch-like ECH-associated protein 1 function in prostate cancer cells causes chemoresistance and radioresistance and promotes tumor growth. Mol. Cancer Ther..

[120] Nioi P., Nguyen T. (2007). A mutation of Keap1 found in breast cancer impairs its ability to repress Nrf2 activity. Biochem. Biophys. Res. Commun..

[121] Konstantinopoulos P.A., Spentzos D., Fountzilas E., Francoeur N., Sanisetty S., Grammatikos A.P., Hecht J.L., Cannistra S.A. (2011). Keap1 mutations and Nrf2 pathway activation in epithelial ovarian cancer. Cancer Res.

[122] Kim Y.R., Oh J.E., Kim M.S., Kang M.R., Park S.W., Han J.Y., Eom H.S., Yoo N.J., Lee S.H. (2010). Oncogenic NRF2 mutations in squamous cell carcinomas of oesophagus and skin. J. Pathol..

[123] Miyamoto N., Izumi H., Miyamoto R., Kondo H., Tawara A., Sasaguri Y., Kohno K. (2011). Quercetin induces the expression of peroxiredoxins 3 and 5 via the Nrf2/NRF1 transcription pathway. Invest. Ophthalmol. Vis. Sci..

[124] Kelly D.P., Scarpulla R.C. (2004). Transcriptional regulatory circuits controlling mitochondrial biogenesis and function. Genes Dev..

[125] Li R., Luciakova K., Nelson B.D. (1996). Expression of the human cytochrome c1 gene is controlled through multiple Sp1-binding sites and an initiator region. Eur. J. Biochem..

[126] Deng Y.R., Chen X.J., Chen W., Wu L.F., Jiang H.P., Lin D., Wang L.J., Wang W., Guo S.Q. (2019). Sp1 contributes to radioresistance of cervical cancer through targeting G2/M cell cycle checkpoint CDK1. Cancer Manag. Res..

[127] Span P.N., Bussink J. (2015). Biology of Hypoxia. Semin. Nucl. Med..

[128] Semenza G.L. (2003). Targeting HIF-1 for cancer therapy, Nature reviews. Cancer.

[129] Marampon F., Gravina G.L., Zani B.M., Popov V.M., Fratticci A., Cerasani M., Di Genova D., Mancini M., Ciccarelli C., Ficorella C., Di Cesare E., Festuccia C. (2014). Hypoxia sustains glioblastoma radioresistance through ERKs/DNA-PKcs/HIF-1α functional interplay. Int. J. Oncol..

[130] Semenza G.L. (2009). Regulation of cancer cell metabolism by hypoxia-inducible factor 1. Semin. Cancer Biol..

[131] Yang W., Wei J., Guo T., Shen Y., Liu F. (2014). Knockdown of miR-210 decreases hypoxic glioma stem cells stemness and radioresistance. Exp. Cell Res..

[132] Grosso S., Doyen J., Parks S.K., Bertero T., Paye A., Cardinaud B., Gounon P., Lacas-Gervais S., Noel A., Pouyssegur J., Barbry P., Mazure N.M., Mari B. (2013). MiR-210 promotes a hypoxic phenotype and increases radioresistance in human lung cancer cell lines. Cell Death Dis..

[133] Feng H., Wang J., Chen W., Shan B., Guo Y., Xu J., Wang L., Guo P., Zhang Y. (2016). Hypoxia-induced autophagy as an additional mechanism in human osteosarcoma radioresistance. J. Bone Oncol..

[134] Zou Y.M., Hu G.Y., Zhao X.Q., Lu T., Zhu F., Yu S.Y., Xiong H. (2014). Hypoxia-induced autophagy contributes to radioresistance via c-Jun-mediated Beclin1 expression in lung cancer cells. J. Huazhong Univ. Sci. Technol. Med. Sci..

[135] Sun Y., Xing X., Liu Q.I., Wang Z., Xin Y., Zhang P., Hu C., Liu Y. (2015). Hypoxia-induced autophagy reduces radiosensitivity by the HIF-1α/miR-210/Bcl-2 pathway in colon cancer cells. Int. J. Oncol..

[136] Buckley A.M., Lynam-Lennon N., O'Neill H., O'Sullivan J. (2020). Targeting hallmarks of cancer to enhance radiosensitivity in gastrointestinal cancers. Nat. Rev. Gastroenterol. Hepatol..

[137] Storozhuk Y., Hopmans S.N., Sanli T., Barron C., Tsiani E., Cutz J.C., Pond G., Wright J., Singh G., Tsakiridis T. (2013). Metformin inhibits growth and enhances radiation response of non-small cell lung cancer (NSCLC) through ATM and AMPK. Br. J. Cancer.

[138] Feng T., Li L., Ling S., Fan N., Fang M., Zhang H., Fang X., Lan W., Hou Z., Meng Q., Jin D., Xu F., Li Y. (2015). Metformin enhances radiation response of ECa109 cells through activation of ATM and AMPK. Biomed. Pharmacother..

[139] Fasih A., Elbaz H.A., Hüttemann M., Konski A.A., Zielske S.P. (2014). Radiosensitization of pancreatic cancer cells by metformin through the AMPK pathway. Radiat. Res..

[140] Jin H., Gao S., Guo H., Ren S., Ji F., Liu Z., Chen X. (2016). *Re*-sensitization of radiation resistant colorectal cancer cells to radiation through inhibition of AMPK pathway. Oncol Lett.

[141] Jeong Y.K., Kim M.S., Lee J.Y., Kim E.H., Ha H. (2015). Metformin Radiosensitizes p53-Deficient Colorectal Cancer Cells through Induction of G2/M Arrest and Inhibition of DNA Repair Proteins. PLoS ONE.

[142] Griss T., Vincent E.E., Egnatchik R., Chen J., Ma E.H., Faubert B., Viollet B., DeBerardinis R.J., Jones R.G. (2015). Metformin antagonizes cancer cell proliferation by suppressing mitochondrial-dependent biosynthesis. PLoS Biol..

[143] Benej M., Hong X., Vibhute S., Scott S., Wu J., Graves E., Le Q.T., Koong A.C., Giaccia A.J., Yu B., Chen C.S., Papandreou I., Denko N.C. (2018). Papaverine and its derivatives radiosensitize solid tumors by inhibiting mitochondrial metabolism. Proc. Natl. Acad. Sci. U S A.

[144] Zhao H., Jiang H., Li Z., Zhuang Y., Liu Y., Zhou S., Xiao Y., Xie C., Zhou F., Zhou Y. (2017). 2-Methoxyestradiol enhances radiosensitivity in radioresistant melanoma MDA-MB-435R cells by regulating glycolysis via HIF-1alpha/PDK1 axis. Int. J. Oncol..

[145] Shen H., Hau E., Joshi S., Dilda P.J., McDonald K.L. (2015). Sensitization of glioblastoma cells to irradiation by modulating the glucose metabolism. Mol. Cancer Ther..

[146] Buckley A.M., Dunne M.R., Lynam-Lennon N., Kennedy S.A., Cannon A., Reynolds A.L., Maher S.G., Reynolds J.V., Kennedy B.N., O'Sullivan J. (2019). Pyrazinib (P3), [(E)-2-(2-Pyrazin-2-yl-vinyl)-phenol], a small molecule pyrazine compound enhances radiosensitivity in oesophageal adenocarcinoma. Cancer Lett..

[147] Fukuda K., Sakakura C., Miyagawa K., Kuriu Y., Kin S., Nakase Y., Hagiwara A., Mitsufuji S., Okazaki Y., Hayashizaki Y., Yamagishi H. (2004). Differential gene expression profiles of radioresistant oesophageal cancer cell lines established by continuous fractionated irradiation. Br. J. Cancer.

[148] Candas D., Lu C.L., Fan M., Chuang F.Y., Sweeney C., Borowsky A.D., Li J.J. (2014). Mitochondrial MKP1 is a target for therapy-resistant HER2-positive breast cancer cells. Cancer Res..

